# Effects of Three Sweet Potato Varieties on the Development, Survival, and Reproduction of *Spodoptera frugiperda* Based on an Age-Stage, Two-Sex Life Table Analysis

**DOI:** 10.3390/insects17050514

**Published:** 2026-05-19

**Authors:** Zhao Wang, Guy Smagghe, Guiyun Long, Huan Zhu, Shaozhao Qin, Zhuting Zhang, Lingling Li

**Affiliations:** 1 College of Life and Health Science, Kaili University, Kaili 556011, China; hdwangzhao@126.com (Z.W.); ab1985916@126.com (H.Z.); sz_qin@163.com (S.Q.); zhangzhuting120@163.com (Z.Z.); 2 Institute of Entomology, Guizhou University, Guiyang 550025, China; guysma9@gmail.com; 3 Department of Biology, Vrije Universiteit Brussel (VUB), 1050 Brussels, Belgium; 4 School of Ethnic Medicine, Guizhou Minzu University, Guiyang 550025, China; 5 Guizhou Key Laboratory of Agricultural Biosecurity, Institute of Plant Protection, Guizhou Academy of Agricultural Sciences, Guiyang 550006, China; lilingling2019@163.com

**Keywords:** fall armyworm, host adaptability, life span, fecundity, population growth parameters, host transfer

## Abstract

To examine how host plant identity shapes the performance of the invasive pest *Spodoptera frugiperda* (J.E. Smith), three sweet potato varieties (Qianshu 12, Qianshu 17, and Yushu 13) were evaluated under laboratory conditions, with maize (Xida 818) as a control. Development, survival, reproduction, and population parameters were assessed using an age-stage, two-sex life table approach. The results demonstrated that *S. frugiperda* completed its life cycle on all sweet potato varieties. The developmental period was significantly different in the larval stage. *S. frugiperda* fed with Yushu 13 had the longest pupal duration (9.97 d) and preadult duration (37.94 d). Reproductive performance was also reduced on sweet potato: adults reared on maize showed greater longevity and higher fecundity, whereas no significant differences were found between Qianshu 17 and Yushu 13. Survival patterns differed among host plants and developmental stages. Early instar survival was highest on Qianshu 12 but lowest on Yushu 13, and Qianshu 12 supported relatively higher survival from late larval stages to adulthood. Life table analysis further showed that larvae fed with three sweet potato varieties exhibited a lower net reproductive rate (*R*_0_), intrinsic rate of increase (*r*), finite rate of increase (*λ*), and longer mean generation time (*T*) compared to those fed with corn. Overall, although sweet potato can support the development of *S. frugiperda*, it constrains reproduction and population growth, indicating lower host suitability.

## Simple Summary

*Spodoptera frugiperda* (fall armyworm) is a well-known invasive pest that damages many crops, especially maize. This study looked at whether it can also thrive on sweet potato. Three sweet potato varieties (Qianshu 12, Qianshu 17, and Yushu 13) were tested and compared with maize (Xida 818). Our results showed that the insect can survive and complete its life cycle on all three sweet potato types. However, it grows more slowly and produces far fewer offspring than when feeding on maize. Overall, sweet potato is a much less suitable host, meaning it is unlikely to support serious pest outbreaks. These findings can help guide practical pest management in the field.

## 1. Introduction

*Spodoptera frugiperda* (J.E. Smith) (Lepidoptera: Noctuidae), originally distributed throughout the tropical and subtropical regions of the Americas, is a major agricultural pest distinguished by its capacity for seasonal long-distance migration and its ability to inflict extensive crop losses [1,2]. Over the past decade, this species has expanded rapidly beyond its native range, colonizing Africa, Oceania, Europe, and much of Southeast Asia [3,4,5,6]. Its arrival in China in late 2018 marked a significant turning point; since then, infestations have damaged crops across more than 1.32 million hectares [7,8,9,10]. Such rapid geographic expansion highlights not only its dispersal ability but also its ecological plasticity in novel environments.

A defining biological feature of *S. frugiperda* is its exceptionally broad host range. The species is reported to utilize over 350 plant species belonging to 76 families, with a marked preference for Poaceae, followed by Asteraceae and Fabaceae [11]. Despite this generalist tendency, populations are structured into two host-associated strains distinguished by feeding preference and genetic markers: the “corn strain”, which primarily exploits crops such as *Zea mays* and *Sorghum bicolor*, and the “rice strain”, which favors *Oryza sativa* and certain forage grasses [12,13,14]. Molecular evidence based on COI fragment alignment and TPi haplotype analysis indicates that the invasive populations in China belong predominantly to the corn strain [15].

Host association in this species is not merely behavioral but has measurable consequences for life-history performance. Larvae of the corn strain feeding on maize typically exhibit shorter developmental times, higher survival rates, and increased adult fecundity compared with those reared on alternative hosts, resulting in more rapid population growth [16,17]. At the same time, laboratory and field studies consistently demonstrate that *S. frugiperda* retains considerable flexibility in host use [18,19]. When preferred resources are scarce or when population density rises, individuals readily shift to secondary crops or even non-cultivated plants. This host-switching capacity is a key ecological trait, enabling persistence under fluctuating conditions and sustaining continuous pressure on agricultural systems [20,21,22].

Since its invasion of China, *S. frugiperda* has been the focus of extensive research addressing its biology, monitoring, and control [23,24,25]. Within this body of work, host adaptability remains central, as it directly informs both risk assessment and the development of targeted management strategies. Previous studies indicate that the species performs well not only on staple crops such as maize, wheat, and sweet potato, but also on economically important plants including soybean and various vegetables [10,26,27]. However, much of this knowledge is based on limited host comparisons, and systematic evaluations across crop varieties remain relatively scarce.

Sweet potato [*Ipomoea batatas* (L.) Lam.] is a globally important tuber crop and ranks among the major staples following rice, wheat, maize, and cassava [28]. China is both the largest producer and consumer of sweet potatoes. According to statistical data of the sweet potato industry, China’s sweet potato growing area in 2025 achieved 0.87 million ha with an annual output value of about 23 billion CNY [29,30]. Our previous laboratory studies revealed that the invasive corn strain of *S. frugiperda* on sweet potato had a smaller net reproductive rate, lower intrinsic rate of increase and finite rate of increase compared to maize and soybean. However, it was capable of completing its life cycle [10]. Nevertheless, detailed assessments of host suitability, particularly across different cultivars, are still lacking. This gap is important, as varietal differences in plant traits may influence larval performance, survival, and reproductive output, thereby affecting pest population dynamics.

The age-stage, two-sex life table provides a robust framework for investigating such questions. Unlike traditional life tables that focus solely on females or average age classes, this method incorporates variability among individuals and developmental stages, as well as both sexes. As a result, it allows for more accurate estimation of key demographic parameters, including survival rate, fecundity, intrinsic rate of increase, and net reproductive rate [31,32,33]. These parameters are essential for evaluating host suitability and predicting population growth under different environmental conditions. Accordingly, the method has been widely applied in studies of *S. frugiperda* to assess host plant effects and varietal resistance [34,35].

In China, most life table analyses of *S. frugiperda* have focused on maize, rice, and vegetable crops, with comparatively little attention given to sweet potato cultivars. Given the crop’s economic importance and the pest’s demonstrated capacity for host expansion, a more systematic evaluation is warranted. The present study examines the developmental duration, survivorship, and fecundity of *S. frugiperda* reared on three representative sweet potato varieties (Qianshu 12, Qianshu 17, and Yushu 13), with maize (Xida 818) used as a control. By constructing age-stage, two-sex life tables, we quantify key population parameters and compare the relative suitability of these hosts. The underlying hypothesis is that sweet potato cultivars differ in their capacity to support population growth of the corn strain, and that such differences can be detected through life table analysis. The results are intended to provide a clearer biological basis for assessing risk and improving management strategies for this invasive pest.

## 2. Materials and Methods

### 2.1. Tested Plants

Seeds of corn (Xida 818; Chongqing Shengqing Seed Industry Co., Ltd., Chongqing, China) were purchased from the local market. Seedlings of three sweet potato varieties (Qianshu 12, Qianshu 17, and Yushu 13) were provided by the Institute of Biotechnology, Guizhou Academy of Agricultural Sciences. All host plants were cultivated in the Botanical Garden of Kaili University under natural environmental conditions, without the application of chemical fertilizers or insecticides. Fresh, fully expanded leaves were collected as food for the insects.

### 2.2. Insect Source

Larvae of *S. frugiperda* were collected from maize fields in Gechong Village, Kaili City, Guizhou, China, in 2023. The insects were subsequently maintained in an artificial climate incubator under controlled laboratory conditions: 25 ± 1 °C, 70 ± 5% relative humidity (RH), and a photoperiod of 16 h light followed by 8 h darkness. The colony was reared separately on each host plant variety for four successive generations to allow acclimation. Eggs from the fifth generation were then used for the experimental trials.

### 2.3. Experimental Methods

#### 2.3.1. Determination of Growth and Development Parameters of *S. frugiperda*

For each host plant, at least 200 eggs laid by the laboratory population were collected. After hatching, neonate larvae of the same age cohort were individually transferred into 12-well plastic culture dishes, with each larva representing one biological replicate. Fresh leaves from each plant variety (Xida 818, Qianshu 12, Yushu 13, and Qianshu 17) were provided as food, with ten replicates established per treatment. The culture dishes were supplied with newly collected leaves daily to ensure continuous food availability. The developmental duration and survival status of each individual were recorded every day. Upon pupation, food provision was discontinued, and pupal weight was measured.

Following adult emergence, individuals were sexed and recorded. Newly emerged males and females were paired and transferred into transparent disposable plastic cups (height: 77 mm, bottom diameter: 47 mm, mouth diameter: 66 mm), each clearly labelled. A cotton ball soaked in 10% honey solution was provided as a food source for the adults, and the cup opening was covered with sterile gauze secured by rubber bands. Adult longevity and daily fecundity (number of eggs laid) were recorded, and the honey solution was replenished daily using a syringe.

All experiments were conducted under controlled environmental conditions of 25 ± 1 °C, 70 ± 5% RH, and a 16:8 h light:dark photoperiod.

#### 2.3.2. Construction of the Age-Stage, Two-Sex Life Table of *S. frugiperda*

Based on the growth and developmental parameters described in Section 2.3.1, including developmental duration, pupal weight, adult longevity, and fecundity, the age-stage, two-sex life table of *S. frugiperda* was constructed in accordance with established theoretical frameworks [36,37,38].

### 2.4. Data Analysis

All raw data were recorded in Microsoft Excel 2003 (Microsoft, Redmond, WA, USA). Statistical analyses were conducted using SPSS 13.0 software (IBM Inc., Armonk, NY, USA) to calculate the mean and standard error (SE) for each parameter. Differences among treatments were evaluated by one-way analysis of variance (ANOVA). All figures were generated using OriginPro 2025 software (OriginLab Corp., Northampton, MA, USA).

#### 2.4.1. Age-Stage-Specific Survival Rate of *S. frugiperda*

According to the age-stage, two-sex life table constructed in Section 2.3.2, the age-stage-specific survival rate (*S_xj_*) describes the probability that an individual *Spodoptera frugiperda* survives from the egg stage to a given age (*x*) and developmental stage (*j*).

#### 2.4.2. Age-Specific Survival Rate and Fecundity of *S. frugiperda*

The age-specific survival rate (*l*_x_) $l_{x}=\sum_{j=1}^{k}S_{xj}$ refers to the probability that an individual survives from the egg stage to age *x*, regardless of stage differentiation, where *j* and *k* represent developmental stages.

Female age-stage-specific fecundity (*f_x__j_*) denotes the number of eggs laid by a female adult at age *x* and stage *j*.

Population age-specific fecundity (*m_x_*) $m_{x}=\sum_{j=1}^{m}S_{xj}f_{xj}/\sum_{j=1}^{m}S_{xj}$ is the average number of eggs laid by the population at age *x*. Since reproductive capacity only occurs in female adults (stage 10) of *S. frugiperda*, the f_10_ curve is presented in the figures of this study.

The population age-specific net reproductive rate (*l_x_m_x_*) is the product of the age-specific survival rate (*l_x_*) and the population age-stage-specific fecundity (*m_x_*).

#### 2.4.3. Reproductive Value of *S. frugiperda*

Reproductive value (*v_xj_*) describes the contribution of an individual at age *x* and stage *j* to future population growth, $v_{xj}=\frac{e^{r(x+1)}}{S_{xj}}\sum_{i=x}^{\alpha }e^{-r(i+1)}\sum_{y=j}^{k}{S\prime }_{ij}f_{ij}$, where *r* is the intrinsic rate of increase and $f_{iy}$ denotes the number of eggs laid at age x and stage y.

#### 2.4.4. Life Expectancy of *Spodoptera frugiperda*

Life expectancy (*e_xj_*) represents the number of additional days that an individual at age *x* and stage *j* is expected to survive, i.e., $e_{xj}=\sum_{i=x}^{\alpha }\sum_{y=j}^{k}{S^{\prime }}_{ij}$, the ${S^{\prime }}_{ij}$ probability that an individual at age *x* and stage *j* survives to age *i* and stage *y*.

#### 2.4.5. Population Dynamic Parameters of *Spodoptera frugiperda*

Based on the age-stage, two-sex life table constructed in Section 2.3.2, key population dynamic parameters of *S. frugiperda* were calculated, including the intrinsic rate of increase (*r*, defined as the maximum population growth potential under favorable environmental conditions) $\sum_{x=0}^{\infty }e^{-r(x+1)}l_{x}m_{x}=1$, $R_{0}=\sum_{x=0}^{\infty }l_{x}m_{x}$ net reproductive rate (*R*_0_), $\lambda =e^{r}$ finite rate of increase (*λ*), and $T=\frac{\ln R_{0}}{r}$ mean generation time (*T*).

## 3. Results

### 3.1. Effects of Different Sweet Potato Varieties on Growth and Development Parameters of S. frugiperda

Statistical analysis of the experimental data indicated that larvae of *Spodoptera frugiperda* fed on the three sweet potato varieties (Qianshu 12, Qianshu 17, and Yushu 13) were able to successfully complete their life cycle, in comparison with those reared on corn (Xida 818). However, the total developmental duration of sweet potato was extended by approximately 7–9 days relative to corn (Table 1). No significant differences were detected among the three sweet potato varieties in terms of overall developmental time.

At the stage-specific level, the larval period (1st to 6th instars) and prepupal stage were significantly prolonged compared with the control (corn, Xida 818) (*p* < 0.05, Table 1). Although the three sweet potato hosts exerted varying effects on individual larval instars, these differences did not translate into significant variation across the entire pre-adult period. Notably, the pupal duration of *S. frugiperda* reared on Yushu 13 (9.97 ± 0.14 d) was significantly longer than that observed on Qianshu 12 (9.42 ± 0.12 d) and Qianshu 17 (9.46 ± 0.17 d).

### 3.2. Effect of Different Sweet Potato Varieties on Reproduction Parameters of S. frugiperda

The reproductive parameters of *S. frugiperda* reared on four host varieties (Xida 818, Qianshu 12, Qianshu 17, and Yushu 13) are presented in Table 2. Adult longevity, for both males and females, was greatest on Xida 818 and shortest on Qianshu 17. No significant differences were observed in the adult pre-oviposition period (APOP) among the four host treatments. In contrast, the total pre-oviposition period (TPOP) varied among hosts, being shortest on Xida 818 and longest on Yushu 13 and Qianshu 17, with no significant difference between the latter two. The longest oviposition period was recorded on Xida 818 (7.06 d), while the shortest periods occurred on Qianshu 17 (4.81 d) and Yushu 13 (5.16 d). The mean fecundity of females on Xida 818 was 1000, which was significantly higher than that on Qianshu 12 (484), Qianshu 17 (357) and Yushu 13 (292).

### 3.3. Effect of Different Sweet Potato Varieties on Survival Rate of S. frugiperda

**Figure 1:**
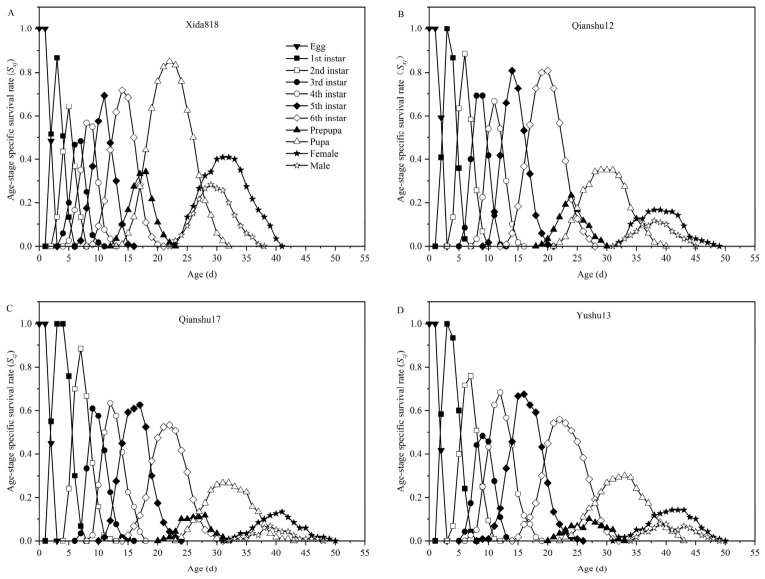
Age-stage specific survival rate (*s_xj_*) of *Spodoptera frugiperda* fed with corn (**A**) and three sweet potato varieties (**B**–**D**).

Using corn (Xida 818) as the control, analysis of the age-stage-specific survival rates (*S_xj_*) of *S. frugiperda* (Figure 1) revealed clear differences among populations reared on the three sweet potato varieties (Qianshu 12, Qianshu 17, and Yushu 13). The survival curves of different developmental stages showed marked overlap, reflecting the well-known asynchrony in insect development, where individuals within the same cohort progress through stages at different rates.

During the early larval period, survival varied substantially among host plants. The survival rates of 2nd and 3rd instar larvae fed on Qianshu 12 reached 88.58% and 69.17%, respectively, representing the highest survival among the sweet potato treatments at this stage. These values were comparable to those observed on the control (Xida 818), both maintaining relatively high survival and significantly exceeding those recorded on the other two varieties. Larvae reared on Qianshu 17 showed intermediate performance, with survival rates of 88.37% (2nd instar) and 60.83% (3rd instar). In contrast, individuals feeding on Yushu 13 exhibited the lowest survival in early instars, with only 75.83% survival in the 2nd instar and 48.33% in the 3rd instar, indicating greater early-stage mortality.

This pattern shifted in later developmental stages, including the 4th to 6th instar larvae, prepupal, pupal, and adult phases. Among all treatments, *S. frugiperda* reared on Qianshu 12 displayed the highest survival rates across these later stages, suggesting greater developmental stability and supporting longer adult longevity. Survival of individuals on Yushu 13 during the later larval stages was slightly lower than that on Qianshu 12, while survival during the prepupal, pupal, and adult stages was intermediate between Qianshu 12 and Qianshu 17. By contrast, Qianshu 17 consistently showed the lowest survival across all subsequent stages among the sweet potato varieties, with cumulative mortality occurring throughout development and resulting in the poorest overall survival.

Under all sweet potato treatments, a clear sex-specific pattern emerged in adult survival. Female adults consistently exhibited higher survival rates than males. This difference was most pronounced in the Qianshu 12 group, where the female survival curve declined more gradually, indicating extended longevity. In comparison, male adults showed shorter lifespans and more rapid mortality, highlighting a consistent female survival advantage across host plants.

### 3.4. Effect of Different Sweet Potato Varieties on Fertility of S. frugiperda

**Figure 2:**
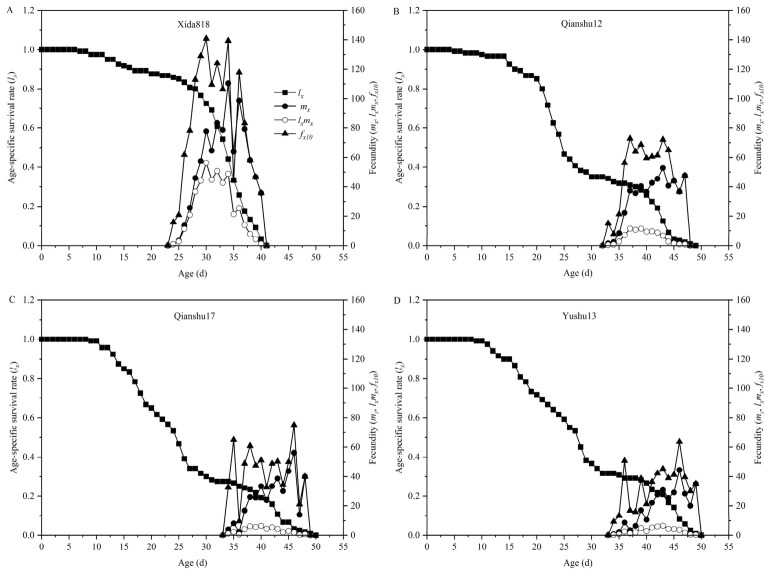
Age-specific survival rate (*l_x_*) and fecundity of *Spodoptera frugiperda* fed with corn (**A**) and three sweet potato varieties (**B**–**D**).

Significant differences were observed in the age-specific survival rate (*l_x_*) and fecundity (*f_x10_*) of *S. frugiperda* when reared on different sweet potato varieties (Figure 2). Individuals maintained on Xida 818 showed relatively high survival during the early stages but exhibited a comparatively short lifespan (38 d). In contrast, those reared on Qianshu 12 displayed the longest overall survival period (49 d), characterized by a gradual decline in survival from 0 to 22 d, followed by a more rapid decrease between 23 and 27 d.

For larvae fed on Qianshu 17 and Yushu 13, survival began to decline after approximately 8 d and decreased to around 30% by 28 d. The decline was more gradual in the Yushu 13 treatment, suggesting slightly better maintenance of survivorship relative to Qianshu 17. Both treatments ultimately reached zero survival after 50 d, indicating similar maximum lifespan limits under these host conditions.

Fecundity patterns (*f_x10_*) further reflected host plant effects on reproductive output. For *S. frugiperda* reared on Qianshu 12, Qianshu 17, and Yushu 13, peak fecundity occurred between 30 and 45 d, but the magnitude of these peaks was markedly lower than that observed on Xida 818. Among the three sweet potato varieties, Qianshu 17 produced a slightly higher fecundity peak than the others; however, all sweet potato treatments showed delayed and reduced reproductive output, with peak *f_x10_* values remaining around 140, in contrast to the higher peak recorded on Xida 818 (occurring at 28–35 d). These patterns suggest that while sweet potato can support complete development of *S. frugiperda*, it imposes constraints on both survival dynamics and reproductive performance compared with the maize host.

### 3.5. Effect of Different Sweet Potato Varieties on Life Expectancy of S. frugiperda

**Figure 3:**
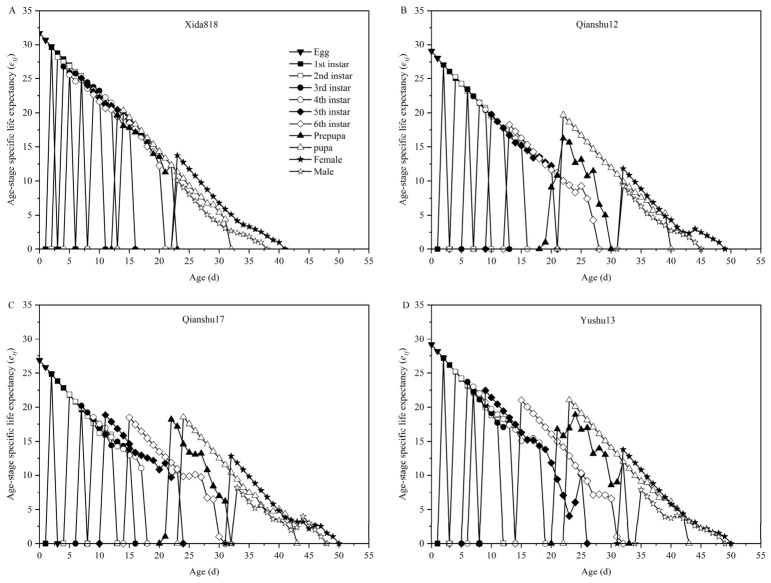
Age-stage specific life expectancy (*e_xj_*) of *Spodoptera frugiperda* fed with corn (**A**) and three sweet potato varieties (**B**–**D**).

The age-stage-specific life expectancy (*e_xj_*) of *S. frugiperda* at each developmental stage showed a gradual decline with increasing age when individuals were reared on the different sweet potato varieties (Figure 3). The life expectancy of newly laid eggs can be regarded as an indicator of the average potential lifespan of the population. Among the sweet potato treatments, it was highest for individuals reared on Yushu 13 (29.18), followed closely by Qianshu 12 (29.05), and lowest on Qianshu 17 (26.85). However, all three values remained lower than the life expectancy of newly laid eggs for individuals fed on Xida 818 (approximately 32), indicating a generally reduced longevity potential on sweet potato compared with corn.

Across developmental stages, the life expectancy of larvae decreased progressively with instar advancement. A similar declining trend was observed for prepupae, pupae, and both male and female adults, with *e_xj_* values continuing to decrease as age increased. Differences in adult life expectancy among host treatments were consistent with the patterns observed in average adult longevity. Specifically, adult life expectancy in the Yushu 13 and Qianshu 12 treatments was significantly higher than that recorded in the Qianshu 17 treatment, suggesting that Qianshu 17 imposes greater physiological constraints on adult survival. Overall, these results indicate that host plant quality influences not only developmental performance but also the expected lifespan across successive stages of *S. frugiperda*, with measurable variation among sweet potato cultivars.

### 3.6. Effect of Different Sweet Potato Varieties on Reproductive Value of S. frugiperda

**Figure 4:**
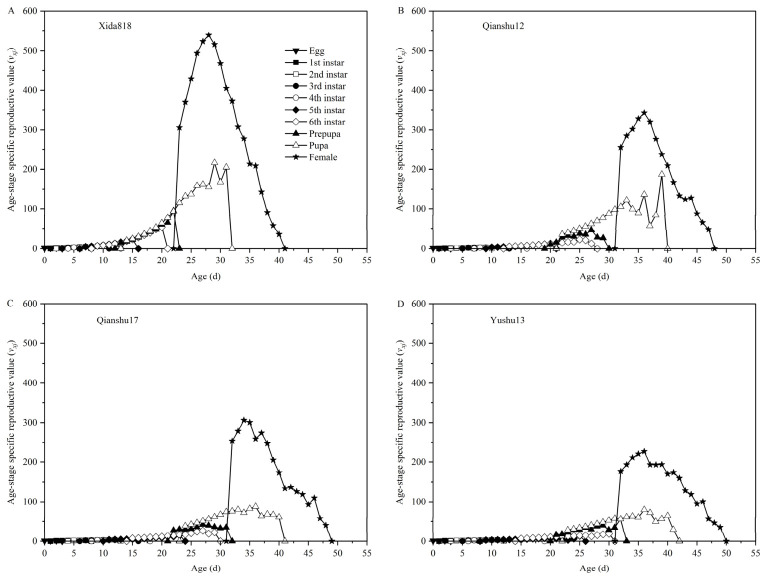
Age-stage specific reproductive value (*v_xj_*) of *Spodoptera frugiperda* fed with corn (**A**) and three sweet potato varieties (**B**–**D**).

The age-stage-specific reproductive value (v_xj_) of S. frugiperda reared on the different sweet potato varieties exhibited a broadly similar unimodal pattern with increasing age (Figure 4). The reproductive values of newly laid eggs for individuals fed on Qianshu 12, Qianshu 17, and Yushu 13 were 1.12, 1.10, and 1.09, respectively. These initial values were comparable to those observed on Xida 818 and increased gradually as individuals developed through successive stages.

For individuals reared on the control host (Xida 818), reproductive value reached a pronounced peak of approximately 550 eggs around day 28. In contrast, those fed on sweet potato varieties reached lower maximum reproductive values at later ages. Individuals reared on Yushu 13 attained their peak reproductive value (227) on day 36, whereas those on Qianshu 12 and Qianshu 17 peaked at day 36 (343) and day 35 (300), respectively.

Overall, the timing of peak reproductive value for *S. frugiperda* on Qianshu 12, Qianshu 17, and Yushu 13 was relatively consistent, occurring within a narrow window of 35–36 d. Among the sweet potato treatments, Qianshu 12 produced the highest peak reproductive value; however, all three sweet potato varieties supported substantially lower peak reproductive outputs compared with Xida 818. These results indicate that while sweet potato hosts can sustain reproduction, they reduce the magnitude of reproductive potential and delay peak reproductive timing relative to maize.

### 3.7. Effect of Different Sweet Potato Varieties on Population Parameters of S. frugiperda

Significant differences were detected in the life table parameters of *S. frugiperda* reared on the different sweet potato varieties (Table 3). The net reproductive rate (*R*_0_), intrinsic rate of increase (*r*), and finite rate of increase (*λ*) for individuals fed on Xida 818 were 433.367 ± 45.454, 0.191 ± 0.004, and 1.211 ± 0.005, respectively. These values were all significantly higher than those recorded for the three sweet potato treatments, indicating a much greater population growth potential on the corn host.

Among the sweet potato varieties, populations reared on Qianshu 12 exhibited *R_0_*, *r*, and *λ* values of 84.775 ± 16.920, 0.111 ± 0.005, and 1.117 ± 0.006, respectively. These parameters were significantly higher than those observed on Qianshu 17 (47.533 ± 11.416, 0.094 ± 0.007, 1.099 ± 0.007) and Yushu 13 (46.267 ± 9.818, 0.090 ± 0.005, 1.095 ± 0.006). No significant differences were detected between Qianshu 17 and Yushu 13 for these population growth indices, suggesting comparable suitability of these two varieties in terms of supporting population increase.

In terms of mean generation time (*T*), individuals reared on Yushu 13 exhibited the longest duration (42.430 ± 0.673 d), followed by those on Qianshu 17 (40.970 ± 0.720 d) and Qianshu 12 (40.122 ± 0.412 d), although no significant differences were observed among the three sweet potato treatments. Notably, the generation time of *S. frugiperda* on all sweet potato varieties was significantly longer than that on corn (Xida 818, 31.754 ± 0.323 d).

Taken together, these results suggest that while sweet potato hosts can support complete development and reproduction of *S. frugiperda*, they generally reduce population growth rates and prolong generation time compared with maize, with Qianshu 12 showing relatively higher suitability among the sweet potato cultivars tested.

## 4. Discussion

Host plants constitute the fundamental biological resource underlying growth, development, reproduction, metabolism, and population dynamics of herbivorous insects. The degree to which an insect can adapt to a given host largely determines its colonization success and outbreak potential [39,40]. As a globally important invasive pest, *S. frugiperda* primarily feeds on gramineous crops such as maize and rice, reflecting its evolutionary adaptation to these hosts. However, its capacity to utilize non-gramineous plants is of increasing ecological and agricultural concern, as such flexibility may expand its host range and complicate management strategies [41]. In the present study, using maize (Xida 818) as a reference, we evaluated the effects of three sweet potato varieties (Qianshu 12, Qianshu 17, and Yushu 13) on the biological performance of *S. frugiperda*, including development, survival, reproduction, and population growth parameters derived from life table analysis. From the perspective of host-insect interactions and nutritional ecology, the results provide insight into the suitability of sweet potato as an alternative host and its implications for pest risk assessment and integrated pest management (IPM).

The results demonstrated that *S. frugiperda* was capable of completing its entire life cycle on all three sweet potato varieties; however, developmental duration was extended by 7–9 days compared with individuals reared on maize. Significant differences were mainly observed in the larval and prepupal stages relative to the control, while variation among sweet potato varieties was less pronounced, with the exception of pupal duration, which was longest on Yushu 13. These findings are consistent with earlier studies reporting prolonged immature development of *S. frugiperda* on alternative hosts such as sweet potato, soybean, and peanut compared with maize [9]. From a physiological perspective, maize provides a more favorable nutritional profile and contains relatively lower levels of defensive secondary metabolites. In contrast, sweet potato leaves contain higher concentrations of polyphenols, flavonoids, crude fiber, and other defensive compounds, which can adversely affect herbivore performance [42,43]. In addition, the physical structure of sweet potato foliage may reduce feeding efficiency.

When feeding on sweet potato, larvae likely experience increased detoxification demands, requiring activation of metabolic pathways involved in the degradation of plant allelochemicals. This process diverts energy away from growth and development, resulting in delayed molting, extended larval duration, and slower progression to pupation [44]. Such trade-offs between detoxification and growth are commonly observed in herbivorous insects exposed to suboptimal or chemically defended host plants.

Reproductive performance is closely linked to host plant quality, particularly the availability of key nutrients required for ovarian development and egg production [45]. In this study, adult longevity, oviposition period, and fecundity followed a consistent ranking across hosts, with maize (Xida 818) supporting the highest values, followed by Qianshu 12, and then Qianshu 17 and Yushu 13. In contrast, the total pre-oviposition period showed the opposite trend. These differences reflect variation in host nutritional suitability. Successful reproduction in *S. frugiperda* depends on sufficient intake of soluble carbohydrates, amino acids, sterols, and mineral nutrients, all of which contribute to vitellogenesis and reproductive maturation [46]. Maize leaves are relatively rich in these components, facilitating rapid adult maturation, extended oviposition periods, and high fecundity.

By comparison, sweet potato tissues contain lower levels of readily available soluble sugars and proteins, with a higher proportion of starch and structural carbohydrates such as fiber [47]. These characteristics reduce digestibility and nutrient assimilation efficiency. As a consequence, larvae feeding on sweet potato may fail to accumulate adequate reserves for optimal reproductive development, leading to reduced egg production and shorter oviposition periods. This nutritional limitation provides a mechanistic explanation for the observed reductions in reproductive output on sweet potato hosts.

Host plant defensive traits also play a critical role in determining insect survival across developmental stages. In this study, age-stage-specific survival analyses revealed clear differences in early larval survival among host plants, followed by stage-dependent shifts in survival patterns during later development. Notably, survival was higher in females than in males across treatments. Early instar larvae are particularly sensitive to host quality due to their limited detoxification capacity and weaker digestive systems, making them vulnerable to plant secondary metabolites and physical barriers [48].

The relatively high survival of early instars on Qianshu 12, comparable to that on maize, suggests that this variety may be more suitable for early larval establishment than the other sweet potato cultivars. As larvae develop, their physiological capacity for detoxification improves, enabling them to better tolerate plant defensive compounds. Consequently, survival during later stages becomes more dependent on nutritional adequacy rather than toxicity. This stage-specific shift highlights the dynamic interaction between insect physiology and host plant characteristics [48,49].

Population growth parameters derived from life tables provide an integrated assessment of host suitability [50]. In the present study, maize supported significantly higher net reproductive rate (*R_0_*), intrinsic rate of increase (*r*), and finite rate of increase (*λ*), along with a shorter mean generation time (*T*), compared with the sweet potato varieties. These parameters indicate a substantially higher population growth potential on maize, consistent with its status as a preferred host. Among the sweet potato varieties, Qianshu 12 supported relatively higher population growth parameters, while Qianshu 17 and Yushu 13 exhibited similar and lower values, with longer generation times.

Higher values of *r* and *R_0_* are generally associated with increased population fitness and a greater likelihood of outbreak formation under favorable environmental conditions [40,51]. From this perspective, maize clearly represents the most suitable host for *S. frugiperda*, enabling rapid population expansion. In contrast, sweet potato imposes constraints on population growth by reducing reproductive output and prolonging generation time. Although the pest can complete its life cycle on sweet potato, its population growth rate is significantly suppressed, reducing the probability of large-scale outbreaks under equivalent conditions.

Despite these insights, several limitations should be acknowledged. First, the study was conducted under controlled laboratory conditions, which do not fully capture the complexity of field environments, including climatic variability, natural enemies, and plant phenological changes. These factors may influence host suitability and insect performance in ways not reflected in laboratory assays. Second, only three sweet potato varieties were evaluated, and their representativeness may not encompass the full genetic and phenotypic diversity of sweet potato cultivars. Additional varieties with different morphological and biochemical traits should be assessed to generalize the findings. Third, the study focused primarily on nutritional and demographic parameters, while underlying physiological and molecular mechanisms, such as detoxification enzyme activity, gene expression related to metabolism, and plant secondary metabolite profiles, were not directly examined. Therefore, we suggest that future research should integrate multi-level approaches, combining life table analysis with biochemical, physiological, and molecular investigations to better understand host–insect interactions. Field-based validation is also necessary to confirm whether the patterns observed under laboratory conditions translate to natural agroecosystems. Furthermore, evaluating the role of environmental stressors, such as temperature fluctuations and interspecific competition, would provide a more comprehensive assessment of pest dynamics on alternative hosts. Expanding the range of sweet potato cultivars and identifying resistant traits could also contribute to breeding programs aimed at enhancing crop resistance.

In summary, the present study demonstrates that while sweet potato can serve as a developmental host for *S. frugiperda*, it is significantly less suitable than maize in terms of development, survival, reproduction, and population growth. Among the tested varieties, Qianshu 12 shows relatively higher suitability, whereas Qianshu 17 and Yushu 13 impose stronger constraints on population performance. These findings improve our understanding of host adaptability in *S. frugiperda*, highlight the ecological consequences of host switching, and provide a scientific basis for developing IPM strategies and resistant sweet potato cultivars in agricultural systems.

## 5. Conclusions

*S.frugiperda* is able to complete its entire life cycle on the three sweet potato varieties (Qianshu 12, Qianshu 17, and Yushu 13), indicating a certain capacity to utilize sweet potato as an alternative host. However, compared with corn, individuals reared on sweet potato exhibited prolonged developmental duration, reduced fecundity, and significantly lower population growth rates, reflecting decreased overall fitness. These constraints suggest that sweet potato is a less suitable host and is unlikely to support large-scale population outbreaks under field conditions. Among the tested varieties, Qianshu 12 showed relatively higher suitability and therefore a slightly greater potential risk of infestation, whereas Qianshu 17 and Yushu 13 demonstrated stronger resistance to *S. frugiperda*.

## Figures and Tables

**Table 1:** Developmental period and total longevity of *Spodoptera frugiperda* fed with corn and three sweet potato varieties.

Stages	Corn Variety		Sweet Potato Varieties					
	*n*	Xida818	*n*	Qianshu12	*n*	Qianshu17	*n*	Yushu13
Egg (d)	120	2.48 ± 0.05 a	120	2.59 ± 0.05 a	120	2.45 ± 0.05 a	120	2.42 ± 0.05 a
1st instar (d)	120	2.02 ± 0.06 c	119	2.67 ± 0.06 b	120	3.67 ± 0.06 a	120	3.40 ± 0.08 a
2nd instar (d)	120	1.71 ± 0.05 b	118	2.58 ± 0.06 a	118	3.04 ± 0.08 a	120	2.80 ± 0.07 a
3rd instar (d)	120	1.52 ± 0.05 c	117	2.49 ± 0.06 a	114	2.33 ± 0.05 ab	115	1.99 ± 0.05 bc
4th instar (d)	117	2.07 ± 0.06 c	116	2.49 ± 0.06 bc	98	2.95 ± 0.08 b	105	3.65 ± 0.10 a
5th instar (d)	115	2.78 ± 0.07 c	104	4.04 ± 0.08 b	73	4.55 ± 0.10 b	76	5.21 ± 0.12 a
6th instar (d)	111	3.93 ± 0.07 c	64	6.59 ± 0.13 b	45	6.42 ± 0.13 b	48	7.42 ± 0.20 a
Prepupa (d)	104	1.77 ± 0.07 b	43	2.16 ± 0.07 a	33	1.94 ± 0.06 ab	38	1.63 ± 0.08 b
Pupa (d)	92	8.58 ± 0.11 c	38	9.42 ± 0.12 b	26	9.46 ± 0.17 b	34	9.97 ± 0.14 a
Preadult (d)	92	26.95 ± 0.23 b	38	35.18 ± 0.34 a	26	37.00 ± 0.50 a	34	37.94 ± 0.51 a
Adult (d)	92	8.27 ± 0.20 a	38	7.63 ± 0.28 b	26	6.85 ± 0.36 c	34	7.03 ± 0.35 c
Total longevity (d)	92	35.22 ± 0.32 b	38	42.82 ± 0.42 a	26	43.85 ± 0.57 a	34	44.97 ± 0.47 a

Values (mean ± SE) followed by different lowercase letters within the same row were significantly different at *p* < 0.05 when analyzed using the one-way ANOVA.

**Table 2:** Reproductive parameters of *Spodoptera frugiperda* fed with corn and three sweet potato varieties.

Parameters	Corn Variety		Sweet Potato Varieties					
	*n*	Xida818	*n*	Qianshu12	*n*	Qianshu17	*n*	Yushu13
Male longevity (d)	40	6.45 ± 0.13 a	17	6.00 ± 0.19 a	10	4.80 ± 0.20 c	15	5.00 ± 0.20 bc
Female longevity (d)	52	9.67 ± 0.17 a	21	8.95 ± 0.19 bc	16	8.12 ± 0.24 d	19	8.63 ± 0.23 cd
APOP (d)	52	1.65 ± 0.08 a	21	1.67 ± 0.11 a	16	1.56 ± 0.13 a	19	2.00 ± 0.15 a
TPOP (d)	52	28.75 ± 0.32 c	21	36.57 ± 0.43 b	16	38.25 ± 0.64 a	19	39.21 ± 0.62 a
Oviposition period (d)	52	7.06 ± 0.15 a	21	6.10 ± 0.12 b	16	4.81 ± 0.19 d	19	5.16 ± 0.16 cd
Fecundity (eggs/female)	52	1000 ± 14 a	21	484 ± 9 b	16	357 ± 22 c	19	292 ± 6 c

Values (mean ± SE) followed by different lowercase letters within the same row were significantly different at *p* < 0.05 when analyzed using the one-way ANOVA.

**Table 3:** Population parameters of *Spodoptera frugiperda* fed with corn and three sweet potato varieties.

Hosts	Varieties	Parameters			
		Net Reproductive Rate (*R_0_*)	Intrinsic Rate of Increase (*r*, Day^−1^)	Finite Rate of Increase (*λ*, Day^−1^)	Mean Generation Time (*T*, Day)
Corn	Xida818	433.367 ± 45.454 a	0.191 ± 0.004 a	1.211 ± 0.005 a	31.754 ± 0.323 b
Sweet potato	Qianshu12	84.775 ± 16.920 b	0.111 ± 0.005 b	1.117 ± 0.006 b	40.122 ± 0.412 a
	Qianshu17	47.533 ± 11.416 c	0.094 ± 0.007 c	1.099 ± 0.007 c	40.970 ± 0.720 a
	Yushu13	46.267 ± 9.818 c	0.090 ± 0.005 c	1.095 ± 0.006 c	42.430 ± 0.673 a

Values (mean ± SE) followed by different lowercase letters within the same column were significantly different at *p* < 0.05 when analyzed using the one-way ANOVA.

## Data Availability

The original contributions presented in this study are included in the article. Further inquiries can be directed to the corresponding author.
